# Current status and future challenges of CAR-T cell therapy for osteosarcoma

**DOI:** 10.3389/fimmu.2023.1290762

**Published:** 2023-12-22

**Authors:** Shizhe Li, He Zhang, Guanning Shang

**Affiliations:** ^1^ Department of Orthopaedics, Shengjing Hospital of China Medical University, Shenyang, Liaoning, China; ^2^ Department of Orthopaedics, The First Affiliated Hospital of Jinzhou Medical University, Jinzhou, Liaoning, China

**Keywords:** CAR-T, osteosarcoma, immunotherapy, targeted therapy, tumor microenvironment

## Abstract

Osteosarcoma, the most common bone malignancy in children and adolescents, poses considerable challenges in terms of prognosis, especially for patients with metastatic or recurrent disease. While surgical intervention and adjuvant chemotherapy have improved survival rates, limitations such as impractical tumor removal or chemotherapy resistance hinder the treatment outcomes. Chimeric antigen receptor (CAR)-T cell therapy, an innovative immunotherapy approach that involves targeting tumor antigens and releasing immune factors, has shown significant advancements in the treatment of hematological malignancies. However, its application in solid tumors, including osteosarcoma, is constrained by factors such as low antigen specificity, limited persistence, and the complex tumor microenvironment. Research on osteosarcoma is ongoing, and some targets have shown promising results in pre-clinical studies. This review summarizes the current status of research on CAR-T cell therapy for osteosarcoma by compiling recent literature. It also proposes future research directions to enhance the treatment of osteosarcoma.

## 1 Introduction

Osteosarcoma, the most common primary malignant bone tumor in children and adolescents, presents significant therapeutic challenges in terms of improving overall patient survival (1). While the combination of surgical resection and adjuvant chemotherapy has contributed to better outcomes in primary osteosarcoma, with a five-year survival rate of approximately 70% (2), recurrent or metastatic osteosarcoma poses greater difficulties. Factors such as chemotherapy resistance and the inability to surgically remove the tumor contribute to a much lower overall survival rate of approximately 25% (3). Consequently, there is an urgent need for novel treatment approaches in this context.

**Figure 1:**
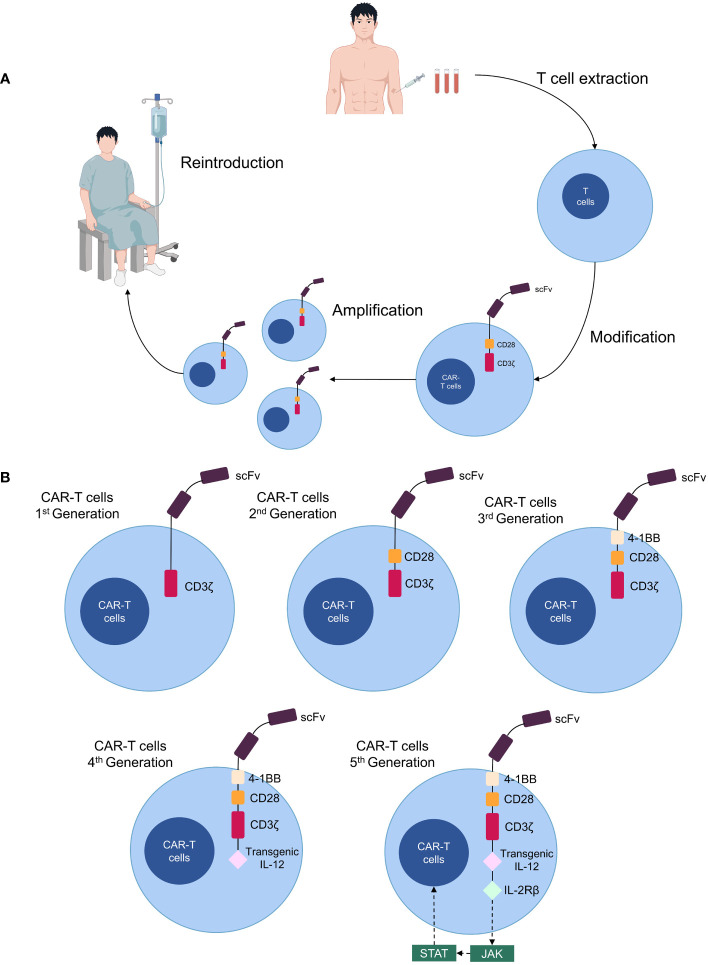
Production processes and structure of CAR-T cells. **(A)** Patient T cells are isolated from the blood and transformed into CAR-T cells *in vitro*, expanded, then cultured and transfused back for therapy. **(B)** Five generations of CAR-T cell structure.

Chimeric antigen receptor (CAR)-T cell therapy is a novel form of immunotherapy derived from adoptive T cell transfer therapy (4). CARs are synthetic receptors composed most often of an extracellular single-chain fragment variable that recognizes tumor antigens, an intracellular signaling domain consisting of the T cell receptor and CD3ζ chain, a transmembrane structural domain, and an extracellular spacer region that adjusts the distance between CAR-T cells and the tumor (5, 6). CAR-T cells are generated by isolating T cells from the patient’s blood, genetically modifying them *in vitro* to express CARs, expanding and culturing them, and subsequently infusing them back into the patient as anti-cancer therapy (7) (**Figure 1A**). Four generations of CAR-T cells exist; the first generation lacks co-stimulatory domains, making them less durable and effective (8). Second-generation CAR-T cells increase their proliferative capacity by adding a co-stimulatory structural domain (CD28, 4-1BB, OX40, etc.); thus, most CAR-T cell therapies currently in clinical use involve second-generation structures (9). The third generation includes additional co-stimulatory molecules compared to the second generation (10), while the fourth generation is further modified to express suicide genes or secrete cytokines (IL-12, IL-15, and IL-21) for enhanced anti-tumor efficacy (11). The concept of a “fifth-generation CAR-T” has also been proposed, involving the addition of an IL-2 receptor beta-chain fragment (IL-2Rβ) to second-generation CAR-T cells, which activates antigen specificity by regulating the JAK/STAT pathway (12, 13) (**Figure 1B**).

Compared to conventional therapies, CAR-T cell therapy is more precise and durable and can secrete cytokines to improve the tumor microenvironment (TME). In recent years, significant progress has been made in the treatment of non-solid tumors (14). However, research is still ongoing for malignant solid tumors such as osteosarcoma, with some targets exhibiting satisfactory results in preclinical trials and substantial potential for clinical applications. This article reviews the potential targets of CAR-T cell therapy in osteosarcoma, summarizes the current status and future directions of CAR-T cell therapy in osteosarcoma, and provides new insights into the treatment of osteosarcoma.

## 2 Potential targets for CAR-T cell-targeted treatment of osteosarcoma

**Figure 2:**
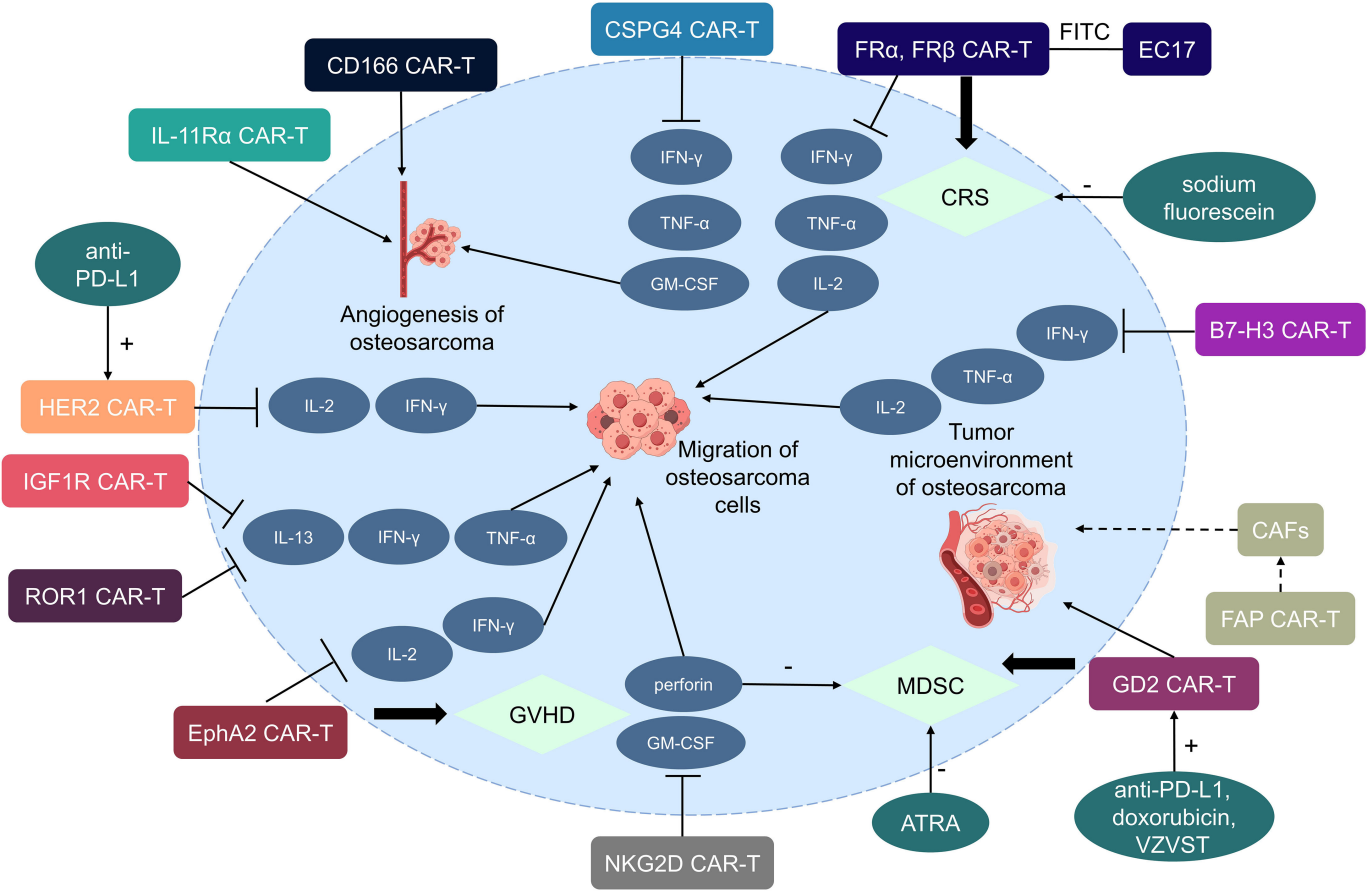
Potential targets for CAR-T cells in the treatment of osteosarcoma.

The majority of potential targets for CAR-T cell therapy in osteosarcoma are tumor-associated antigens, which are expressed in both normal and tumor tissues but are more highly expressed in tumor tissues. Several current potential targets for CAR-T cell therapy in osteosarcoma include receptor tyrosine kinases (HER2, IGF1R, ROR1, and EphA2), cell surface glycoproteins (CSPG4, FRα, FRβ, and EC17), B7-H3 (CD276), disialoganglioside (GD2), natural killer group 2D (NKG2D), activated leukocyte cell adhesion molecule (ALCAM/CD166), interleukin-11 receptor alpha (IL-11Rα), and fibroblast activation protein (FAP) (**Figure 2**, **Table 1**).

**Table 1:** Potential effective targets for CAR-T therapy in osteosarcoma.

Target	Characteristics	Expression in normal tissue	Anti-tumor ability	Disadvantages	Clinical trials
HER2	A receptor tyrosine kinase	Low expression in normal tissue	Kills osteosarcoma cells and metastases	Off-target toxicity	NCT00902044
IGF1R	A receptor tyrosine kinase	Expression in normal tissue	Kills osteosarcoma cells	Off-target toxicity	–
ROR1	A receptor tyrosine kinase	Expression in normal tissue	Kills osteosarcoma cells	Off-target toxicity	–
EphA2	A receptor tyrosine kinase	Low expression in normal tissue	Kills osteosarcoma cells and metastases	GVHD	–
CSPG4	A cell surface glycoprotein	Low expression in normal tissue	Kills osteosarcoma cells	No *in vivo* experiments	–
FR	A cell surface glycoprotein	Low expression in normal tissue	Kills osteosarcoma cells	CRS	–
B7-H3	A member of the B7 family of immunoregulatory proteins	Low expression in normal tissue	Kills osteosarcoma cells and metastases	–	NCT04483778
GD2	A glycolipid antigen containing N-acetylneuramic acid	Expression in GD2^+^ neuronal tissue	Kills osteosarcoma cells	MDSCs	NCT01953900, NCT02107963, NCT03635632
NKG2D	A activating receptor expressed by NK cells and T cells	Expression in NK cells and T cells	Kills osteosarcoma cells	Off-target toxicity and low treatment efficiency	–
ALCAM	A type-I membrane protein	Widely expressed in normal tissue	Kills osteosarcoma cells	Off-target toxicity	–
IL-11Rα	A member of the MAPK, PI3K, and JAK-STAT activating family of cytokines	Low expression in normal tissue	Kills osteosarcoma cells and metastases	Inefficient treatment	–
FAP	A type-II transmembrane proteolytic enzyme	Low expression in normal tissue	–	No CAR-T related studies	–

### 2.1 Receptor tyrosine kinase

Receptor tyrosine kinases play a crucial role in regulating various cellular processes such as tumor cell proliferation, invasion, migration, and differentiation through the modulation of signaling pathways such as PI3K/AKT, Ras/MEK/ERK, and JAK/STAT (15). In osteosarcoma, several receptor tyrosine kinases have the potential for CAR-T cell therapy, including HER2 and IGF1R.

#### 2.1.1 Human epidermal growth factor receptor 2

The epidermal growth factor receptor family comprises four transmembrane receptors: HER1, HER2, HER3, and HER4 (16). Unlike other receptors, HER2 does not require ligand binding for heterodimerization, and its overexpression leads to intracellular domain dimerization and autophosphorylation, activating downstream signaling pathways involved in tumor cell proliferation, invasion, migration, and angiogenesis. Consequently, HER2 exhibits potent oncogenic potential (17). HER2 is frequently overexpressed and associated with poor prognosis in various malignancies, including breast cancer and gastric cancer (18, 19). Recent studies have demonstrated high expression of HER2 in osteosarcoma and its correlation with poor prognosis (20, 21). While the HER2 inhibitor trastuzumab has limited efficacy in metastatic osteosarcoma, HER2 remains a potential target for immunotherapy (22). Research by Ahmed et al. showed that second-generation HER2-targeting CAR-T cells secreted immunostimulatory cytokines, including interferon IFN-γ and interleukin IL-2, upon exposure to HER2-positive osteosarcoma cells, leading to tumor growth inhibition (23). In a xenograft osteosarcoma mouse model, HER2-targeted CAR-T cells induced regression of primary lesions and lung metastases (23). Another study by Rainusso et al. demonstrated that HER2-targeted CAR-T cells reduced the myosphere-forming ability of osteosarcoma cells *in vitro*, suggesting the potential efficacy of HER2-targeted CAR-T cell therapy in treating metastatic osteosarcoma (24). Additionally, Park et al. found that combining HER2 CAR-T cells with PD-L1 antibodies increased T cell expression in an osteosarcoma xenotransplantation model, providing further support for the clinical application of HER2-targeted CAR-T therapy (25). Several ongoing clinical trials are evaluating the use of HER2-targeted CAR-T cells in osteosarcoma treatment. A phase I/II clinical trial (NCT00902044) involving 16 patients with HER2-positive osteosarcoma showed that escalating doses of second-generation CAR-T cells (dose range 1×10^4^ to 1×10^8^/m^2^) resulted in CAR-T cell persistence for at least six weeks. Four patients achieved stable disease for 12 weeks to 14 months, with a median progression-free survival of approximately six weeks. Only one patient experienced fever, and no other severe adverse events were reported (26). Although HER2 is a promising target for CAR-T cells in the treatment of osteosarcoma, its practical clinical applications are limited. Morgan et al. reported a case of severe respiratory failure in a patient with HER2-positive colon cancer after treatment with CAR-T cells targeting HER2 (27). They hypothesized that this may be related to CAR-T cells recognizing HER2 expressed in normal lung tissue and releasing inflammatory factors, which in turn leads to pulmonary toxicity and a cytokine storm (27). Therefore, further studies focusing on the safety aspects of CAR-T cell therapy targeting HER2 are necessary.

#### 2.1.2 Insulin-like growth factor 1 receptor

The insulin-like growth factor 1 receptor (IGF1R), a receptor tyrosine kinase located on the cell membrane, plays a role in the invasion and metastasis of various malignancies (28). In osteosarcoma, IGF1R is overexpressed and contributes to the early development of the disease by activating the PI3K/AKT pathway, while relying on the RAS/MAPK pathway for late lung metastasis (29, 30). Currently, clinical trials investigating IGF1R-targeted therapy for osteosarcoma are underway (31). IGF1R also holds potential for immunotherapy. Huang et al. demonstrated that third-generation CAR-T cells targeting IGF1R released cytokines such as IFN-γ, TNF-α, and IL-13, leading to anti-tumor effects on osteosarcoma cells. In a xenograft mouse model, CAR-T therapy targeting IGF1R inhibited tumor growth and prolonged survival (32). However, the expression of IGF1R in normal tissues (skeletal muscle) poses a challenge in the development of IGF1R-targeted CAR-T therapies, as off-target toxicity remains a concern (33). Further exploration and studies are necessary to address this issue and advance the development of IGF1R-targeted CAR-T therapies for osteosarcoma.

#### 2.1.3 Receptor tyrosine kinase-like orphan receptor 1

The receptor tyrosine kinase-like orphan receptor 1 (ROR1) is a type-I transmembrane glycoprotein that plays a role in cell differentiation and proliferation during embryonic and fetal development (34). In various malignancies, including osteosarcoma, ROR1 is overexpressed and contributes to the promotion of migration by regulating the Wnt signaling pathway (35, 36). A recent study demonstrated that third-generation CAR-T cells targeting ROR1 effectively inhibited osteosarcoma growth both *in vitro* and *in vivo (* 32). However, similar to IGF1R, the expression of ROR1 in multiple normal tissues (parathyroid, pancreatic islets and duodenal mucosa) raises concerns regarding potential off-target toxicity (37). Lee et al. addressed this issue by utilizing the single-chain fragment variable of zilovertamab, a humanized monoclonal antibody, as the antigen recognition domain of CAR-T cells, resulting in improved safety (38). Several clinical trials have been conducted to evaluate ROR1-targeted CAR-T therapy in patients with solid tumors (NCT05274451, NCT05638828, and NCT02706392). However, these trials did not specifically include patients with osteosarcoma (39). Nevertheless, these studies provide valuable insights that can inform future investigations on the application of ROR1-targeted CAR-T therapy for osteosarcoma.

#### 2.1.4 Ephrin type-A receptor 2

Ephrin type-A receptor 2 (EphA2) is a member of the ephrin family of receptors and plays a role in normal embryonic development and kidney function. Its expression is primarily observed in highly proliferative epithelial tissues (40). EphA2 is highly expressed in various malignancies and is implicated in the epithelial-mesenchymal transition in osteosarcoma (41, 42). A study by Hsu et al. demonstrated that second-generation CAR-T cells targeting EphA2 can effectively inhibit osteosarcoma cells by releasing cytokines such as IL-2 and IFN-γ (43). In a mouse model of metastatic osteosarcoma, treatment with EphA2-targeted CAR-T cells resulted in infiltration of the lungs and liver, tumor eradication, and increased survival time (43). Unlike IGF1R and ROR1, EphA2 is expressed at lower levels in normal tissues than in highly proliferative epithelial tissues, which reduces the likelihood of off-target toxicity (44). However, Hsu et al. also reported that some models developed graft-versus-host disease (GVHD) approximately 50 days after CAR-T cell infusion. This observation may be associated with the origin of the T cells used for CAR-T therapy. The study suggested that targeting CAR-T cells with autologous-derived EphA2 may enhance treatment safety (43).

### 2.2 Cell surface glycoproteins

Cell surface glycoproteins are identifiable tumor antigens of CAR-T cells that can assist T cells in tumor inhibition (45). Currently, two glycoproteins (CSPG4 and FR) are potential candidates for CAR-T cell therapy in osteosarcoma.

#### 2.2.1 Chondroitin sulfate proteoglycan 4

Chondroitin sulfate proteoglycan 4 (CSPG4) is a transmembrane glycoprotein that is highly expressed in various malignancies (46). In osteosarcoma, the expression level of CSPG4 is also correlated with poor prognosis (47). The central role of CSPG4 in malignant tumor progression and metastasis, as well as its impact on the tumor microenvironment, suggests its potential as an effective target for tumor immunotherapy (48). Beard et al. found that second-generation CSPG4-targeted CAR-T cells released IFN-γ, TNF-α, and GM-CSF and exerted cytolytic effects on the MG63 cell line (49). The advantages of CSPG4-targeted CAR-T cells for the treatment of solid tumors include the inhibition of peritumoral angiogenesis and improvement of the tumor microenvironment (50). However, no *in vivo* trials have been conducted on CSPG4 in osteosarcoma; therefore, further studies are required to assess its utility.

#### 2.2.2 Folate receptor and EC17

Folate receptor α (FRα) and FRβ are membrane-bound proteins anchored to the extracellular surface using glycosylphosphatidylinositol and are capable of transporting folate across membranes via endocytosis (51). FRα is mainly expressed at low levels in epithelial tissues, whereas FRβ is found in placental and hematopoietic tissues (51). FRα is highly expressed in osteosarcoma, and FRβ+ tumor-associated macrophages have been detected in metastatic osteosarcoma (52, 53). Therefore, FRα and FRβ are potential therapeutic targets for the treatment of osteosarcoma. EC17, a low-molecular-weight ligand, can be stabilized on the surface of tumor cells due to its high affinity for the folate receptor and coupling with fluorescein isothiocyanate (FITC) (54). Amato et al. employed EC17 combined with folate receptors for immunotherapy in patients with metastatic renal cell carcinoma, which achieved a good effect (55). Lu et al. combined EC17 and FITC with folate receptors according to the concept of the CAR-T adaptor molecule to form second-generation FITC-specific CAR-T cells and applied these cells to the HOS osteosarcoma cell line. Both *in vitro* and *in vivo* experiments showed the strong anti-tumor activity of CAR-T cells (56). However, high doses of folate receptor CAR-T cells can lead to severe cytokine release syndrome (CRS), which can be alleviated by the intravenous administration of sodium fluorescein (56). Therefore, the safety of these targets requires further evaluation.

### 2.3 B7-H3

B7-H3 (CD276) is an immune checkpoint of the B7 family of molecules that is involved in the regulation of T cells. It may also inhibit NK cell activation and play a pro-inflammatory role (57). B7-H3 is overexpressed in a variety of malignancies and is associated with tumor proliferation, metastasis, and poor prognosis (58). Studies have shown that B7-H3 may influence tumor progression by regulating the PI3K/AKT/mTOR, JAK2/STAT3, and TLR4/NF-κB pathways (59–61). Lu et al. examined specimens from 35 patients with advanced osteosarcoma and found a positive B7-H3 expression rate of approximately 29%, suggesting the potential of B7-H3 as a target for osteosarcoma immunotherapy (62). Majzner et al. found that third-generation CAR-T cells targeting B7-H3 released IFN-γ, TNF-α, and IL-2 cytokines when co-cultured with osteosarcoma cells, showing effective anti-tumor abilities. In a mouse model of osteosarcoma, the survival time was prolonged by at least five months with CAR-T cell treatment compared to the control group (63). Talbot et al. implanted LM7 osteosarcoma cells expressing firefly luciferase (fLuc) into the tibia of a mouse model and showed that CAR-T cells targeting B7-H3 exhibited potent dose-dependent anti-lung metastatic activity, as detected by bioluminescence imaging (64). Zhang et al. subsequently constructed a third-generation CAR-T cell model targeting B7-H3, which demonstrated good anti-osteosarcoma efficacy in both *in vivo* and *in vitro* assays (65). A phase I clinical trial of second-generation B7-H3-targeted CAR-T cells for the treatment of recurrent osteosarcoma in children and adolescents is currently underway (NCT04483778). Compared to other immunotherapeutic targets, B7-H3 is expressed at lower levels in normal tissues than in malignant tissues and has less potential for off-target toxicity (63). Additionally, B7-H3 not only affects innate and adaptive immunity but also regulates tumor progression through various non-immune signaling pathways (58–61). Therefore, B7-H3 may be an ideal target for CAR-T cell therapy in osteosarcoma.

### 2.4 Disialoganglioside

Disialoganglioside (GD2) is a tumor-associated surface antigen expressed in the fetus and is also present in neural stem cells with multiple biological functions, including signal transduction and cell adhesion (66). As a tumor antigen, GD2 expression is highly specific to tumors and enhances the interaction between tumor cells and extracellular matrix proteins. In fact, GD2 is highly expressed in various malignancies (67). Roth et al. conducted immunohistochemistry on 44 osteosarcoma specimens and revealed high expression of GD2 in osteosarcoma, with higher staining intensity in recurrent osteosarcoma specimens (68). This suggests that GD2 may play a key role in the progression of osteosarcoma and has potential value in targeted therapy. Long et al. demonstrated that third-generation GD2-targeted CAR-T cells significantly inhibited the growth of osteosarcoma cells *in vitro*. However, in a xenograft model, an increase in myeloid-derived suppressor cells (MDSCs) hindered the efficacy of CAR-T cells *in vivo (* 69). They also suggested that MDSCs may inhibit T cell responses by affecting the CD28-containing co-stimulatory domains in CAR-T cells (69). All-trans retinoic acid (ATRA) can be used to inhibit MDSC activity. In xenografted mouse models, the use of ATRA enhanced CAR-T cell efficacy and improved the survival rate of mice (69). Furthermore, Long et al. found that ATRA may enhance efficacy by increasing the expression of glutathione synthase in MDSCs and mediating the neutralization of reactive oxygen species (69, 70). In addition, the combination of GD2-targeted CAR-T cells and PD-L1 antibodies can improve T cell infiltration in osteosarcoma and enhance its efficacy (25). Chulanetra et al. found that the combination of fourth-generation GD2-targeted CAR-T cells and doxorubicin enhanced the efficacy of doxorubicin against osteosarcoma cells (71). Moreover, Tanaka et al. suggested that live attenuated varicella-zoster virus vaccination enhances the therapeutic efficacy of third-generation GD2-targeted CAR-T cells against malignant solid tumors by affecting the TME (NCT01953900) (72). These results indicate that GD2-targeted CAR-T cell therapy may have potential in multiple drug combinations for osteosarcoma, with broad application prospects. However, Chulanetra et al. showed that an increased ratio of tumor cells to CAR-T cells could reduce the durability of CAR-T cell therapy (71). CAR-T cells acting on osteosarcoma cells upregulate PD-L1 and PD-1 expression, which may diminish the effect of GD2-targeted CAR-T cells (71). Therefore, the efficiency of GD2-targeted CAR-T cells must be further explored. Several clinical trials on GD2-targeted CAR-T therapy for relapsed or refractory osteosarcoma are currently underway (NCT01953900, NCT02107963, and NCT03635632).

### 2.5 Natural killer group 2D

Natural killer group 2D (NKG2D) is an activated immune receptor expressed on NK and T cells, playing a role in the regulation of innate and secondary immune responses (73). NKG2D is normally expressed in NK cells, CD8 T cells, γδ T cells, and some CD4 T cells. In humans, NKG2D primarily recognizes ligands such as MHC I chain-related molecules A and B (MICA and MICB) and the UL16 binding protein family (ULBP1-6) (74). These ligands are often overexpressed in malignant tumors, suggesting an association of NKG2D with tumor progression (75). Fernandez et al. found that activated and amplified NK cells could target osteosarcoma cells *in vivo* and *in vitro* through interactions between NKG2D and its ligands, indicating the potential of NKG2D and its ligands as immunotherapeutic targets for osteosarcoma (76). Subsequently, Fernandez et al. demonstrated that third-generation CAR-T cells targeting NKG2D inhibited osteosarcoma cells in both *in vitro* and *in vivo* assays, prolonging the survival time of xenograft mouse models without associated toxic effects (77). Additionally, Parihar et al. suggested that cytotoxic-chain fusion of NKG2D with the T cell receptor could alleviate MDSCs caused by CAR-T treatment, indicating potential therapeutic options for NKG2D in combination with other CAR-T targets (78). Although NKG2D is minimally expressed on normal cell surfaces compared with NK and T cells, off-target toxicity cannot be ruled out. For example, Van Seggelen et al. reported lethal toxicity in NKG2D-targeted CAR-T cells in a mouse model (79). Moreover, Sentman et al. found that the acute toxic response in a mouse model resembled the symptoms of cytokine release syndrome (CRS) observed in patients treated with CAR-T cells, which was associated with two effectors of CAR-T therapy: perforin and GM-CSF (80). However, no adverse reactions associated with CAR-T cells were observed in a phase I clinical trial of NKG2D-targeted CAR-T cells in patients with refractory multiple myeloma (81). Therefore, further investigation is needed to assess the safety of NKG2D as a therapeutic target.

### 2.6 Activated leukocyte cell adhesion molecule

Activated leukocyte cell adhesion molecule (ALCAM), also known as CD166, is a type-I transmembrane immunoglobulin that plays a role in mediating adhesion between homogeneous and heterogeneous cells in the body (82). ALCAM is highly expressed in various tumors and is involved in processes such as tumor angiogenesis and distant metastasis (83, 84). Immunohistochemical staining conducted by Federman et al. revealed positive expression of ALCAM in the cell membrane and cytoplasm of osteosarcoma cells (85). Furthermore, Cardenes et al. demonstrated that ALCAM facilitates the binding and uptake of tumor-derived extracellular vesicles in colorectal and ovarian cancers, thereby promoting the migration of tumor cells (86). These findings suggest that ALCAM plays a role in regulating osteosarcoma metastasis. In the context of immunotherapy, Wang et al. developed second-generation CD166-BBζ-targeted CAR-T cells that exhibited significant cytotoxicity against osteosarcoma cells *in vitro*, with the cytotoxic effect strongly correlated with the expression of CD166 (87). Additionally, *in vivo* studies showed that CAR-T cells targeting CD166 effectively inhibited osteosarcoma growth without causing off-target toxicity (87). However, it should be noted that CD166 is also expressed in normal tissues, such as epithelial cells (88), raising the possibility of off-target toxicity.

### 2.7 Interleukin-11 receptor alpha

Interleukin-11 (IL-11) is a member of the IL-6 family of cytokines and plays a role in various biological processes such as hematopoiesis, bone development, and tissue repair (89). IL-11 exerts its effects through the IL-11 receptor, which consists of interleukin-11 receptor alpha (IL-11Rα) and GP130 receptors, and activates the JAK signaling pathway. IL-11Rα is believed to be involved in tumor proliferation, invasion, and angiogenesis (90). Lewis et al. found that IL-11 and IL-11Rα were specifically upregulated in primary osteosarcoma cells and lung metastases but minimally expressed in normal bone tissue, and that IL-11Rα expression was associated with poor prognosis (91, 92). Huang et al. applied third-generation IL-11Rα-targeting CAR-T cells to osteosarcoma cells and found that CAR-T cells effectively killed tumor cells and inhibited lung metastases, and that the expression level of IL-11Rα was positively correlated with the killing effect (93). In addition to CAR-T cell therapy, Jiang et al. utilized the high expression of IL-11Rα in osteosarcoma to develop a drug delivery system called IL11-PDox. This system involved functionalizing REDOX-sensitive polymersomes with IL-11 ligand and encapsulating the chemotherapy drug Dox. Their findings showed that IL11-PDox enhanced the efficacy of chemotherapy in mouse models of recurrent and metastatic osteosarcoma, suggesting a potential combination strategy of IL-11Rα-targeted therapy with chemotherapy for advanced osteosarcoma (94). Although no toxic reactions were observed in CAR-T trials targeting IL-11Rα, concerns have been raised about the efficiency of IL-11Rα-targeted therapy because of its underexpression in osteosarcoma (93).

### 2.8 Fibroblast activation protein

The high number of cancer-associated fibroblasts (CAFs) in malignant solid tumors provides an ideal microenvironment for the development of tumor cells (95). CAFs play a crucial role in tumors by secreting various cytokines and metabolites that inhibit immune cells and promote tumor invasion and metastasis. One specific protein expressed by CAFs is fibroblast activation protein (FAP), which belongs to the prolyloligopeptidase family and is minimally expressed in normal human tissues (96). It is now recognized as an effective target for CAFs, and an increasing number of studies have shown that targeting FAP can inhibit tumor progression by modulating the TME (97, 98). Wang et al. demonstrated that CAR-T cells targeting FAP could effectively inhibit tumor growth by targeting tumor stromal cells both *in vitro* and *in vivo*, without significant toxicity. This suggests that FAP-targeted CAR-T therapy may be an effective approach for treating malignant solid tumors (99). Additionally, Zhang et al. found that FAP is highly expressed in osteosarcoma and its expression level correlates with poor prognosis (100). Although no studies have investigated FAP-targeted CAR-T therapy in osteosarcoma, FAP remains a potential target for CAR-T therapy in this disease due to its impact on the TME and its high expression level in osteosarcoma.

## 3 Limitations and perspectives of CAR-T cell therapy for osteosarcoma

Research on malignant solid tumors, including osteosarcoma, has been prompted by the excellent performance of CAR-T cells in the treatment of hematological malignancies. Currently, potential effective targets for CAR-T cell therapy in osteosarcoma include HER2, IGF1R, ROR1, EphA2, CSPG4, folate receptor with EC17, B7-H3, GD2, NKG2D, ALCAM/CD166, IL-11Rα, and FAP. Among these targets, HER2, EphA2, B7-H3, and IL-11Rα CAR-T cells warrant further investigation owing to their effectiveness in metastatic osteosarcoma (24, 43, 64, 93). HER2, B7-H3, and GD2 are currently undergoing clinical trials, so they will likely be the first targets used for the clinical treatment of osteosarcoma. Although CAR-T therapy is a promising treatment for osteosarcoma, low antigen specificity, poor durability, numerous side effects, and the complexity of the tumor microenvironment (TME) still limit its widespread application (101). Therefore, scholars have recently proposed counterstrategies to overcome the various limitations of CAR-T therapy (**Table 2**).

**Table 2:** Current limitations and potential counterstrategies of CAR-T cell therapy in osteosarcoma.

Limitations	Potential strategies
Low antigen specificity	• Finding new targets (TSAs) • Novel CAR-T cells • Multi-specific CAR-T cells • Logic gating of CAR-T cells • Binding multiple bispecific junctions
Poor durability	• Optimizing the source of CAR-T cells • CRISPR-Cas9 gene editing of allo CAR-T cells • Large-scale production of allo CAR-T cells from T cell-derived induced pluripotent stem cells • Reducing ex vivo culture time of CAR-T cells • Drug combination applications • PD-L1 inhibitors • Chemotherapy drugs • PI3K inhibitors • HADC inhibitors etc.
Side effects of CAR-T cells	• CRS • Reduction of inflammatory factor release (inhibitors, CRISPR-Cas9 knockdown) • Suicide genes (HSV-TK, iCasp9) • Application of glucocorticoids • GVHD • Infusion of allo CAR-T cells • MDSCs • Infusion of ATRA
Complex tumor microenvironment	• Reducing the amount of suppressive immune factors • Immune factor antibodies (MDSC antibodies, tumor-associated macrophage antibodies, regulatory T cell antibodies) • CRISPR-Cas9 gene editing to knock out immune factor receptors • Fourth-generation CAR-T cells • Drug combination applications • PD-L1 inhibitors • Chemotherapy drugs • Targets that act on the tumor mesenchyme or microvasculature (FAP, CSPG4, etc.) • Optimizing the administration route of CAR-T cells (local injection)

### 3.1 Antigen specificity

Low antigen specificity is a major limitation of CAR-T cell therapy for solid tumors. The ideal targets for CAR-T cells are tumor-specific antigens (TSAs), which are expressed at high levels in tumor tissue but virtually absent in normal tissue (9). In contrast, the majority of CAR-T targets in osteosarcoma are tumor-associated antigens, and differences in expression between tumor-associated antigens and TSAs in normal tissues may lead to severe off-target toxicity and insufficient efficacy (102). Current responses to this limitation include the search for additional TSA targets and the development of novel CAR-T cells. However, TSA targets in osteosarcoma are rare and difficult to identify, making the development of novel CAR-T cells a more feasible solution to address low specificity (101). For example, Park et al. constructed bispecific CAR-T cells targeting both HER2 and GD2 to act on osteosarcoma cells and improve efficacy (25). Bielamowicz et al. developed multi-specific CAR-T cells targeting HER2, IL13Rα2, and EphA2 to enhance antigen specificity in glioblastoma, which could serve as a reference for their application in osteosarcoma (103). Furthermore, the concept of “logic gating” has been proposed, wherein T cells can improve their specificity by expressing both antigens (A and B) or only antigens A or B (104). Srivastava et al. designed a logically gated ROR1-targeted CAR-T cell that reduced off-target toxicity and selectively targeted tumor cells (105). Similarly, Lee et al. suggested that combining CAR-T cells with multiple bispecific junctions (e.g. FITC) could help target multiple antigens and enhance efficacy in heterogeneous solid tumors (106).

### 3.2 Therapeutic durability of CAR-T cells

In addition to specificity, the durability of CAR-T cells impacts their treatment efficacy, as insufficient durability can affect the long-term therapeutic effects of CAR-T cells *in vivo (* 107). Antigen heterogeneity or antigen loss is the main cause of antigen-dependent drug resistance in CAR-T cells (108). Hegde et al. found that when CAR-T cells act on HER2-positive U373 cells (glioblastoma), HER2-negative tumor cells are produced secondarily, which leads to drug resistance in CAR-T cells (109). In addition to the development of multi-specific CAR-T cells mentioned earlier, it is currently believed that genetic manipulation interventions and drug combinations can enhance the durability of CAR-T cells (110). CAR-T cells can be categorized as autologous or allogeneic based on their source. DiNofia et al. discovered that autologous CAR-T cells exhibited significantly greater durability compared to allogeneic CAR-T cells (111). However, the widespread availability of autologous CAR-T cell preparation is limited due to variations in patient conditions. Therefore, allogeneic CAR-T cells may still be a suitable source, considering their short production cycle and lower cost. Furthermore, Dimitri et al. suggested that the use of CRISPR-Cas9 technology could genetically regulate allogeneic CAR-T cells and improve their persistence (112). Van der Stegen et al. found that induced pluripotent stem cells derived from T cells could contribute to the large-scale development of potent allogeneic CAR-T cells (113). Ex vivo culture time also impacts durability in the preparation of CAR-T cells. Ghassemi et al. found that shortening the ex vivo culture time of CAR-T cells reduced differentiation and improved their efficacy in acute lymphoblastic leukemia (114). Reducing ex vivo expansion time enhances CAR-T cell durability. Castella et al. developed ARI-0001, a closed semi-automatic bioreactor system, to expedite CAR-T cell culture for hematological malignancies, with potential implications for solid tumor applications (115). Combination therapy with other drugs is also a viable approach. PD-L1 inhibitors are believed to reverse the rapid depletion of CAR-T cells (25, 116). Chemotherapeutic agents can enhance CAR-T cell trafficking, infiltration, and anti-tumor efficacy in solid tumors; however, the dosage and side effects of these agents must be carefully considered (71, 94, 117). Zheng et al. suggested that PI3K inhibitors could maintain the hypofunctional state of CAR-T cells without affecting T cell expansion, thereby improving their persistence and reducing tumor burden (118). Additionally, Lei et al. found that the histone deacetylase (HDAC) inhibitor SAHA enhances the anti-tumor activity of B7-H3-targeted CAR-T cells in solid tumors (119). Therefore, these drugs have the potential to function as immunomodulators and optimize CAR-T therapies.

### 3.3 Side effects of CAR-T cells

Side effects associated with CAR-T cells in osteosarcoma include CRS, off-target toxicity, GVHD, and increased MDSCs (43, 56, 69). CRS is the most common adverse event following CAR-T cell therapy and is caused by the release of large amounts of inflammatory factors (IL-6, IL-1, GM-CSF, etc.), which can lead to fever and, in severe cases, heart failure and death (120). GVHD and MDSCs are also frequently observed in *in vivo* osteosarcoma trials. Although CAR-T cell therapy for osteosarcoma is still in pre-clinical research, the dangers associated with its side effects should be taken seriously. IL-6 is currently considered a key factor in the development of CRS, and the IL-6 inhibitor tocilizumab has been approved for the treatment of CRS (121). Other studies have shown that IL-1 inhibitors and GM-CSF inhibitors can reduce the incidence of CRS (122, 123). Ghahri-Saremi et al. proposed that CRISPR-Cas9 technology could be used to produce CAR-T cells that knock down inflammatory factors to reduce the incidence of side effects (124). Additionally, introducing suicide genes into CAR-T cells can be an effective approach. Specifically, the expression of suicide genes, such as herpes simplex thymidine kinase (HSV-TK) and induced caspase 9 (iCasp9), can lead to the selective destruction and ablation of CAR-T cells, thus reducing the occurrence of off-target toxicity and side effects (125). Glucocorticoids may also be effective in suppressing CRS due to their ability to inhibit inflammatory responses. However, it should be noted that glucocorticoids can also impact the efficacy of CAR-T cells (126). The current approach to addressing GVHD and increased MDSCs involves the infusion of allogeneic CAR-T cells and the application of ATRA. However, further exploration of additional solutions is warranted.

### 3.4 Tumor microenvironment

The TME refers to the unique environment created by tumor cells, consisting of tumor cells, supporting stromal cells, microvasculature, cytokines, and chemokines (127). The TME contains more suppressive immune factors (MDSCs, tumor-associated macrophages, and regulatory T cells) than normal tissues and can impede T cell activity (128). In addition, the intercellular matrix produced by CAFs in the periphery of the TME and the surrounding microvasculature together form a physical barrier that limits the penetration of CAR-T cells (129). The “chemical barrier” (suppressive immune factors) and “physical barrier” (interstitial cells and microvasculature) both diminish the capacity of CAR-T cells to penetrate and infiltrate, thereby reducing their efficacy. The unique bone microenvironment in osteosarcoma, characterized by a porous extracellular matrix and abundant blood supply, leads to substantial immune cell infiltration, notably tumor-associated macrophages (TAM) and T lymphocytes, fostering an immune-tolerant setting for tumor chemotaxis. This increases the challenge of overcoming the “chemical barrier” (130, 131). One strategy to address the “chemical barrier” is to decrease the presence of suppressive immune factors using immune factor antibodies, such as MDSC antibodies, TAM antibodies, and regulatory T cell antibodies. Another approach involves employing CRISPR-Cas9 gene editing to target immune factor receptors and remodel the TME (104, 132). Fourth-generation CAR-T cells are now considered to play a regulatory role in the TME owing to their ability to secrete cytokines that stimulate the immune response around tumor cells (133). Some researchers have suggested that a combination of immune checkpoint inhibitors (PD-L1 inhibitors) can increase the number of immune checkpoints in T cells, thus reducing immunosuppression (134). Chemotherapeutic agents can also reduce the levels of immune factors in the TME (117). As for the “physical barrier,” targets that act on the tumor mesenchyme or microvasculature (FAP, CSPG4, etc.) may also improve the penetration of CAR-T cells in the TME (135). Additionally, local injection of CAR-T cells increases their concentration and improves their infiltration capacity (136). Furthermore, Li et al. found that the use of porous microneedle patches during local injection could further improve infiltration without a loss of activity, thus providing a reference for practical clinical applications (137).

## 4 Conclusion

CAR-T cell therapy has shown effectiveness against osteosarcoma in pre-clinical studies, and promising results have been observed in clinical trials targeting certain antigens. However, challenges such as low antigen specificity, limited durability, significant side effects, and an unfavorable TME hinder the wider application of CAR-T therapy in osteosarcoma. To overcome these limitations, future advancements may include the development of novel CAR-T cells, combination therapies with drugs, and gene editing techniques, aiming to enhance the efficacy and safety of CAR-T cell therapies for osteosarcoma treatment.

## Author contributions

SL: Software, Writing – original draft. HZ: Funding acquisition, Writing – review & editing. GS: Funding acquisition, Writing – review & editing.
